# Cirrhosis in intrahepatic cholangiocarcinoma: prognostic importance and impact on survival

**DOI:** 10.1186/s12876-023-02710-w

**Published:** 2023-05-13

**Authors:** Nimish Thakral, Teresita Gonzalez, Olger Nano, Sang-Ha Shin, Shenae Samuels, Atif Hussein

**Affiliations:** 1 Department of Hepatology, University of Kentucky, 740 S. Limestone, Medicine Specialties, Kentucky Clinic Wing C, Room 211, Floor, 2, Lexington, KY 40536 USA; 2 Memorial Healthcare System, Hollywood, USA

**Keywords:** Cholangiocarcinoma, Intrahepatic cholangiocarcinoma, Cirrhosis, Prognostic factor, Palliative care

## Abstract

**Context:**

Cholangiocarcinoma (CCA), a malignancy of the biliary tract epithelium is of increasing importance due to its rising incidence worldwide. There is a lack of data on cirrhosis in intrahepatic CCA (iCCA) and how it affects overall survival and prognosis.

**Objectives:**

The primary objective of this study was to examine if there were differences in survival outcomes between iCCA patients with concomitant cirrhosis and those without cirrhosis.

**Methods:**

The National Cancer Database (NCDB) was used to identify and study patients with iCCA from 2004 to 2017. The presence of cirrhosis was defined using CS Site-Specific Factor 2 where 000 indicated no cirrhosis and 001 indicated the presence of cirrhosis. Descriptive statistics were utilized for patient demographics, disease staging, tumor, and treatment characteristics. Kaplan-Meier (KM) method with log-rank test and a multivariate logistic regression model was used to assess if the presence of cirrhosis in iCCA was associated with survival status and long-term survival (60 or more months after diagnosis).

**Results:**

There were 33,160 patients with CCA in NCDB (2004–2017), of which 3644 patients were diagnosed with iCCA. One thousand fifty-two patients (28.9%) had cirrhosis as defined by Ishak Fibrosis score 5–6 on biopsy and 2592 patients (71.1%) did not meet the definition for cirrhosis. Although in univariate analyses using KM/log-rank tests showed a survival advantage for non-cirrhotic patients, there was no statistically significant association found between cirrhosis and survival status (OR = 0.82, *p* = 0.405) or long-term survival (OR = 0.98, *p* = 0.933) when multivariate analysis was used. iCCA patients with cirrhosis and Stage 1 tumor had the highest median OS (132 months) vs 73.7 months in the non-cirrhotic arm, while patients with stage IV disease who had cirrhosis had half the survival time of those without. Our data thus indicates that the presence of cirrhosis is not an independent prognostic factor for survival.

## Introduction

Cholangiocarcinoma (CCA) is defined as an epithelial cell malignancy of the biliary tract that can arise from any location within the biliary tract and maintains markers of cholangiocyte differentiation. CCA is classified based on the anatomical location of origin – Intrahepatic, Perihilar and Extrahepatic [1]. Mixed hepatocelluar-cholangiocellular carcinomas (HC-CCA), also called combined HCC according to the WHO classification, were only recently identified as a subset of cholangiocarcinoma [2, 3]. Most CCAs are adenocarcinomas with varying grades of differentiation [4, 5].

Globally, the incidence of CCA shows a wide geographical variability with East Asia having the highest rates and Western Europe having the lowest rates of the disease. There is considerable variation in the incidence rates intra-nationally within East Asian countries with age adjusted incidence ratio for North-East Thailand being 85/100,000 and for North and Central Thailand being 14.5/100,000 [6]. Within the United States, age-adjusted incidence rates of CCA are lowest in Non-Hispanic Caucasians and Blacks with Hispanics and Asians showing the highest rates [7]. Since 1973, we have witnessed a gradual increase in the incidence of intrahepatic CCA (iCCA) with a concomitant decrease in the incidence of carcinoma of unknown primary (CUP). This is likely due to improvement in molecular profiling and the resultant ability to identify the tissue of origin [8].

As we identify more cases of CCA, the importance of prognostication is of prime importance. Not only does it enhance our knowledge about the disease but also helps in risk stratification for selection of treatment modality. Previous studies have shown a neutrophil/lymphocyte ratio greater than 3, multiple metastatic sites, an intrahepatic primary site and presence of liver metastases, the number of sites of advanced disease, a poor Eastern Cooperative Oncology Group (ECOG) performance status (PS) and elevated levels of alkaline phosphatase (ALP) to be associated with worse survival [9, 10, 11, 12]. The presence of cirrhosis in iCCA as a prognostic factor for OS has been a contested topic, with some studies showing a correlation between cirrhosis and worse OS, while other studies showing no statistically significant difference. Hence, our aim is to investigate, using a larger sample size of iCCA patients, if survival is affected by the presence of cirrhosis.

## Methods

This study was approved as exempt by the Memorial Healthcare System (MHS) Institutional Review Board (MHS.2021.030). The 2004–2017 National Cancer Database (NCDB) was queried for patients with cholangiocarcinoma (CCA). NCDB is a national hospital-based cancer registry that contains de-identified patient level data, provided to Commission on Cancer (CoC)- accredited cancer programs to help investigators advance cancer research, which in 2012–2014, captured 72.5% of the cancer cases in the United States [13]. Patients with CCA were identified by International Classification of Diseases for Oncology, Third Edition (ICD-O-3)-Oncology morphologic codes 8160/3 (bile duct adenocarcinoma), 8161/3(bile duct cystadenocarcinoma), and 8162/3 (Klatskin tumor). iCCA patients were identified by ICD-O-3 morphologic code 8160/3 with topographical codes C220 (liver) and C221(intrahepatic bile duct). Extrahepatic CCA patients were identified by histology codes 8160/3, 8161/3, 8162/3 with topographical codes C239 (gallbladder) or C240 (extrahepatic bile duct) and not included in the study. The ICD-O-3 has two axis, morphological and topographical code. Morphological code describes the cell type or histology of tumor, while topographical code describes the anatomical site of origin. Patients with iCCA were further categorized by the presence of cirrhosis. The presence of cirrhosis was defined using CS Site Specific Factor 2 where 000 indicated no cirrhosis and 001 indicated the presence of cirrhosis.

Descriptive statistics were utilized for patient demographics, disease staging, tumor, and treatment characteristics. The primary objective of this study was to examine if there were differences in survival outcomes between iCCA patients with concomitant cirrhosis and those without cirrhosis. To assess the study’s primary objective- a multivariate logistic regression was employed. Factors found to be significant in Tables 1 and 2 and based expert knowledge was used as covariates in multivariate logistic regression models. Multivariate logistic regression models, adjusting for socio-demographic and clinical characteristics (insurance type, median income quartile, treatment facility, age, sex, race/ethnicity, Charlson-Deyo comorbidity score, timing of first course treatment, grade, pathological stage and tumor size) was then entered into a stepwise backward selection logistic regression model where variables with *p* ≥ 0.20 were removed to develop the final multivariate regression models to be used in the assessment of the presence of cirrhosis and its association with survival outcomes (survival status and long-term survival). In the assessment of survival status, the final multivariate model from stepwise backward selection adjusted for median income quartile, grade, and pathological stage. In the assessment of long-term survival, the final multivariate model from stepwise backward selection adjusted for facility type, grade, and pathological stage. This was then used to assess if the presence of cirrhosis among intrahepatic patients was associated with survival status (where patients who died served as the reference group) and long-term survival (survival of 60 or more months after the date of diagnosis).

**Table 1:** Demographics among intrahepatic CCA patients by cirrhosis status

	No Cirrhosis (%)	Cirrhosis (%)	*P*-Value
Sample Size	2592 (71.1)	1052 (28.9)	NA
Facility Type			
Community Cancer Program	76 (3.0)	33 (3.2)	0.127
Comprehensive Community Program	495 (19.7)	203 (19.8)	
Academic/Research Program	1579 (62.7)	668 (65.3)	
Integrated Network Cancer Program	369 (14.7)	119 (11.6)	
Age, Median (IQR)	66 (57–74)	63 (57–70)	NA
Age Categories			**< 0.001**
Non-Elderly (18–64 years old)	1178 (45.5)	597 (56.8)	
Elderly (≥ 65 years old)	1414 (54.6)	455 (43.3)	
Sex			**< 0.001**
Male	1239 (47.8)	703 (66.8)	
Female	1353 (52.2)	349 (33.2)	
Race			**0.034**
White	2146 (83.6)	878 (84.3)	
Black	223 (8.7)	105 (10.1)	
Asian	156 (6.1)	40 (3.8)	
Other	41 (1.6)	19 (1.8)	
Ethnicity			**< 0.001**
Non-Hispanic	2337 (92.5)	901 (87.7)	
Hispanic	189 (7.5)	127 (12.4)	
Charlson Deyo Co-Morbidity Score			**< 0.001**
0	1722 (66.4)	504 (47.9)	
1	533 (20.6)	239 (22.7)	
2	189 (7.3)	117 (11.1)	
≥ 3	148 (5.7)	192 (18.3)	

Percentages may not add to 100% due to rounding error and missing data

Per NCDB data use agreement, some values are omitted due to having counts of less than 10

*IQR* Interquartile Range (25th Percentile - 75th Percentile), *NA* Not Applicable

Bold font denotes statistical significance at *P* < 0.05

**Table 2:** Disease staging and tumor characteristics among intrahepatic CCA patients by cirrhosis status

	No Cirrhosis (%)	Cirrhosis (%)	*P*-Value
Diagnosis Year			0.884
2004–2010	534 (20.6)	219 (20.8)	
2011–2017	2058 (79.4)	833 (79.2)	
Grade			**0.007**
Well differentiated (I)	132 (8.6)	69 (12.9)	
Moderately differentiated (II)	813 (53.1)	246 (46.1)	
Poorly differentiated (III)	569 (37.2)	212 (39.7)	
Undifferentiated (IV)	17 (1.1)	(1.3)	
Clinical Stage			0.410
0	(0.0)	(0.1)	
1	552 (28.3)	223 (28.3)	
2	364 (18.6)	140 (17.8)	
3	187 (9.6)	66 (8.4)	
4	851 (43.6)	357 (45.4)	
Pathological Stage			**0.013**
0	(0.5)	(0.0)	
1	295 (28.9)	77 (30.3)	
2	205 (20.1)	50 (19.7)	
3	139 (13.6)	16 (6.3)	
4	378 (37.0)	111 (43.7)	
Bone Metastases			0.280
None	835 (94.9)	321 (93.3)	
Yes; Distant Bone Metastases	45 (5.1)	23 (6.7)	
Lung Metastases			0.778
None	796 (90.8)	314 (91.3)	
Yes; Distant Lung Metastases	81 (9.2)	30 (8.7)	
Other Metastases			0.939
None	793 (90.2)	310 (90.1)	
Yes; Distant Metastases	85 (9.7)	34 (9.9)	
Tumor Size			**< 0.001**
0–2.4 cm	205 (10.0)	122 (15.5)	
2.5–4.9 cm	571 (27.8)	281 (35.8)	
5.0–8.4 cm	726 (35.3)	248 (31.6)	
Equal to or greater than 8.5 cm	553 (26.9)	135 (17.2)	
Lymphovascular Invasion			**0.001**
Not Present	606 (63.3)	179 (74.6)	
Present	351 (36.7)	61 (25.4)	

Percentages may not add to 100% due to rounding error and missing data

Per NCDB data use agreement, some values are omitted due to having counts of less than 10

*NA* Not Applicable

Bold font denotes statistical significance at *P* < 0.05

Overall survival was defined as the time (in months) between the date of diagnosis and date of death or censored at last contact. Kaplan-Meier (KM) method with log-rank test was also used to compare and estimate overall survival rates between iCCA patients with concomitant cirrhosis and those without cirrhosis, as well as stratified by surgical intervention and tumor stage. Statistical significance was defined as *p* < 0.05. Palliative Care was defined as any care provided to palliate or alleviate symptoms, such as surgery, radiation therapy, systemic therapy (chemotherapy, hormone therapy, or other systemic drugs), and/or other pain management therapy. All analyses were conducted using Stata (version 15.1, StataCorp, College Station, Texas).

## Results

**Fig. 1:**
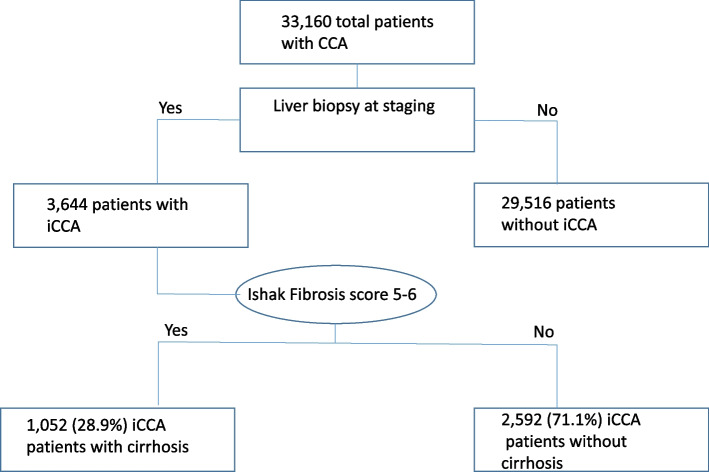
Flow chart detailing the total patients with CCA, and the included patients with iCCA based on cirrhosis status

A total of 3644 patients with iCCA underwent biopsy during the NCDB study period of 2004–2017. iCCA patients with known CS Site Specific Factor 2, 1052 patients (28.9%) had cirrhosis as defined by Ishak Fibrosis score 5–6 on biopsy and 2592 patients (71.1%) did not meet the definition for cirrhosis (Fig. 1).

### Demographics in iCCA patients by cirrhosis status

Table 1 presents demographics of iCCA patients by cirrhosis status. Most patients were seen at an academic/research program. Patients with cirrhosis presented at a younger age (median age: 63 years vs. 66 years) and had a higher percentage of male patients (66.8% vs 47.8%, *p* < 0.001). Both groups mostly included White (84.3% vs 83.6%, *p* = 0.034) and non-Hispanic patients. A higher proportion of patients with cirrhosis were Hispanic [12.4 vs 7.5%, *p* < 0.001)]). Cirrhotic patients also had a greater percentage of patients with three or more comorbidities at the time of diagnosis as defined by a Charlson Devo Co-Morbidity Score (18.3% vs 5.7%, *p* < 0.001).

### Disease staging and tumor characteristics in iCCA patients by cirrhosis status

Table 2 presents disease staging, grade, and tumor characteristics based on cirrhosis status. Most patients were diagnosed between the years of 2011 to 2017. Statistically significant differences were found for grade, pathologic stage, tumor size and lymphovascular invasion. Cirrhotic patients were more likely to have a higher pathological grade at presentation with more patients presenting with poorly differentiated (Grade III) or undifferentiated (Grade IV) cancer as opposed to non-cirrhotic patients (38.3% vs 41.0%, *p* < 0.001). Similarly, there was a higher percentage of cirrhotic patients with pathologic stage 4 disease (43.7% vs 37.0%, *p* = 0.013). Regarding tumor size, cirrhotic patients were more likely to have tumor size less than 5 cm compared to non-cirrhotics (51.3% vs 37.8%, *p* < 0.001). Lymphovascular invasion was lower for cirrhotic iCCA patients than non-cirrhotic iCCA patients; (25.4% vs 36.7%, *p* = 0.001).

### Treatment characteristics in iCCA patients by cirrhosis status

Table 3 presents treatment characteristics among iCCA patients by cirrhosis status. Overall, non-cirrhotic patients were more likely to undergo surgical intervention at primary site (44.9% vs. 31.7%, *p* < 0.001) and chemotherapy (52.3% vs. 44.1%, *p* < 0.001) compared to the cirrhotic cohort. However, both cohorts had similar proportions of patients who received radiation therapy. Compared to non-cirrhotic patients, a higher percentage of cirrhotic iCCA patients were not offered surgery as a part of the first planned treatment compared to non-cirrhotics (56.4% vs 48.0%, *p* < 0.001). Moreover, cirrhotics were more likely to not receive any treatment compared to non-cirrhotics (16.6% vs 12.6%, *p* = 0.004). Cirrhotics also had a higher percentage of patients having to wait longer than 60 days until first surgical procedure (30.3% vs 22.4%, *p* < 0.001), a lower percentage of patients without residual tumor after resection (20.2% vs 32.7%, *p* < 0.001) and a higher contraindication due to patient risk factors to undergo surgery (10.7% vs 6.4%, *p* < 0.001). Cirrhotics were more likely to not receive chemotherapy during treatment, and when chemotherapy was administered, cirrhotics were less likely to receive multiagent chemotherapy (27.0% vs 37.0% *p* < 0.001). No statistically significant difference in palliative care utilization was seen for cirrhotics compared to non-cirrhotics with majority of patients not receiving palliative care, 89.3% for the non-cirrhotic group and 90.1% for the cirrhotic group.

**Table 3:** Treatment characteristics among intrahepatic CCA patients by cirrhosis status

	No Cirrhosis (%)	Cirrhosis (%)	*P*-Value
N (%)	2592 (71.1)	1052 (28.9)	NA
Surgical Intervention Received	1157 (44.9)	331 (31.7)	**< 0.001**
Radiation Therapy Received	444 (17.4)	182 (17.6)	0.871
Chemotherapy Received	1319 (52.3)	453 (44.1)	**< 0.001**
Treatment Status			**0.004**
No Treatment Given	271 (12.6)	145 (16.6)	
Treatment Given	1876 (87.0)	722 (82.5)	
Active Surveillance	(0.5)	(0.9)	
Days from Diagnosis to First Course Treatment, Median (IQR)	36 (20–57)	40 (20–70)	NA
Days from Diagnosis to First Course Treatment			**< 0.001**
Within 30 Days	828 (40.5)	279 (38.3)	
Between 30 and 60 Days	759 (37.1)	229 (31.4)	
Longer than 60 Days	459 (22.4)	221 (30.3)	
**Surgery**			
Days from Diagnosis to First Surgical Procedure, Median (IQR)	40 (19–71)	44 (0–93)	NA
Days from Diagnosis to First Surgical Procedure			**0.002**
Within 30 Days	452 (38.1)	141 (41.2)	
Between 30 and 60 Days	364 (30.7)	72 (21.1)	
Longer than 60 Days	371 (31.3)	129 (37.7)	
Surgical Margins After Resection			**< 0.001**
No Residual Tumor	848 (32.7)	212 (20.2)	
Residual Tumor, NOS	66 (2.6)	20 (1.9)	
Microscopic Residual Tumor	158 (6.1)	36 (3.4)	
Macroscopic Residual Tumor	(0.4)	(0.3)	
Margins Not Evaluable	36 (1.4)	30 (2.9)	
No Primary Site Surgery	1432 (55.3)	721 (68.5)	
Unknown or Not Applicable	42 (1.6)	30 (2.9)	
Reason for No Surgery of Primary Site			**< 0.001**
Surgery Performed	1157 (44.8)	331 (31.6)	
Surgery not a Part of the Planned First Treatment	1240 (48.0)	590 (56.4)	
Contraindication Due to Patient Risk Factors	164 (6.4)	112 (10.7)	
Patient Died Prior to Planned or Recommended Surgery	(0.1)	(0.6)	
Surgery not Performed but Recommended by Physician	(0.0)	(0.3)	
Patient/Family Member/Guardian Refusal	14 (0.5)	(0.3)	
Unknown if Surgery Performed	(0.2)	(0.1)	
**Radiation**			
Sequencing of Radiation and Surgical Procedures			**0.009**
None	2367 (92.0)	988 (94.9)	
Radiation Therapy Before Surgery	44 (1.7)	17 (1.6)	
Radiation Therapy After Surgery	159 (6.2)	36 (3.5)	
Radiation Therapy Both Before and After Surgery	(0.0)	(0.0)	
Intraoperative Radiation Therapy	(0.0)	(0.0)	
Intraoperative Radiation Therapy with Other Therapy Administered Before/After	(0.0)	(0.0)	
Days from Diagnosis to Radiation Therapy, Median (IQR)	81 (47–138)	78 (46–122)	
Days from Diagnosis to Radiation Therapy			0.603
Within 30 Days	46 (10.6)	23 (12.9)	
Between 30 and 60 Days	112 (25.9)	41 (22.9)	
Longer than 60 Days	275 (63.5)	115 (64.3)	
**Systemic**			
Days from Diagnosis to Systemic Therapy, Median (IQR)	49 (29–82)	48 (28–83)	NA
Days from Diagnosis to Systemic Therapy			0.958
Within 30 Days	336 (26.5)	114 (26.0)	
Between 30 and 60 Days	425 (33.5)	145 (33.1)	
Longer than 60 Days	509 (40.1)	179 (40.9)	
Chemotherapy Status and Type			**< 0.001**
Not Administered	1202 (47.7)	574 (55.9)	
First Course, NOS	59 (2.3)	18 (1.8)	
Single Agent Chemo	328 (13.0)	157 (15.3)	
Multi Agent Chemo	932 (37.0)	278 (27.1)	
Days from Diagnosis to Chemotherapy, Median (IQR)	49 (29–82)	48 (28–84)	NA
Days from Diagnosis to Chemotherapy			0.934
Within 30 Days	336 (26.6)	115 (26.2)	
Between 30 and 60 Days	423 (33.4)	144 (32.8)	
Longer than 60 Days	506 (40.0)	180 (41.0)	
Immunotherapy			0.099
Not Administered	1202 (95.3)	574 (97.0)	
Yes; First Course Treatment	59 (4.7)	18 (3.0)	
Days from Diagnosis to Immunotherapy, Median (IQR)	80 (56–184)	142 (88–147)	NA
Days from Diagnosis to Immunotherapy			0.547
Within 30 Days	(8.3)	(20.0)	
Between 30 and 60 Days	(25.0)	(0.0)	
Longer than 60 Days	(66.7)	(80.0)	
Palliative Care			0.479
Yes	277 (10.7)	104 (9.9)	
No	2315 (89.3)	947 (90.1)	

Percentages may not add to 100% due to rounding error and missing data

Per NCDB data use agreement, some values are omitted due to having counts of less than 10

*IQR* Interquartile Range (25th Percentile - 75th Percentile), *NA* Not Applicable

Bold font denotes statistical significance at *P* < 0.05

### Cirrhosis and its association with survival outcomes

There were 3030 observations with cirrhosis status data for which survival information was present. Specifically, survival information was missing for 606 patients of the study population resulting in missing median survival. Furthermore, 8 patients had an elapsed time from the date of initial diagnosis to the date of last contact or death of 0 months and as such, could not be accounted for. Hence, the median OS was not calculated for a total of 614 patients.

**Fig. 2:**
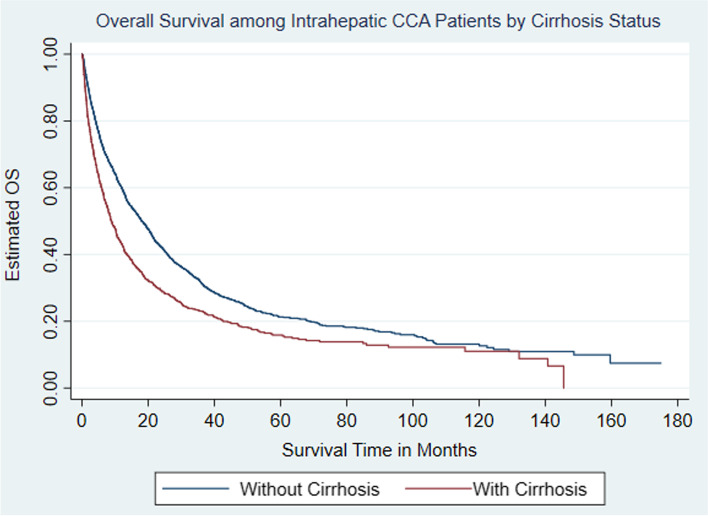
Kaplan-Meier curve estimated Overall Survival (OS) among intrahepatic cholangiocarcinoma (iCCA) by cirrhosis status

**Fig. 3:**
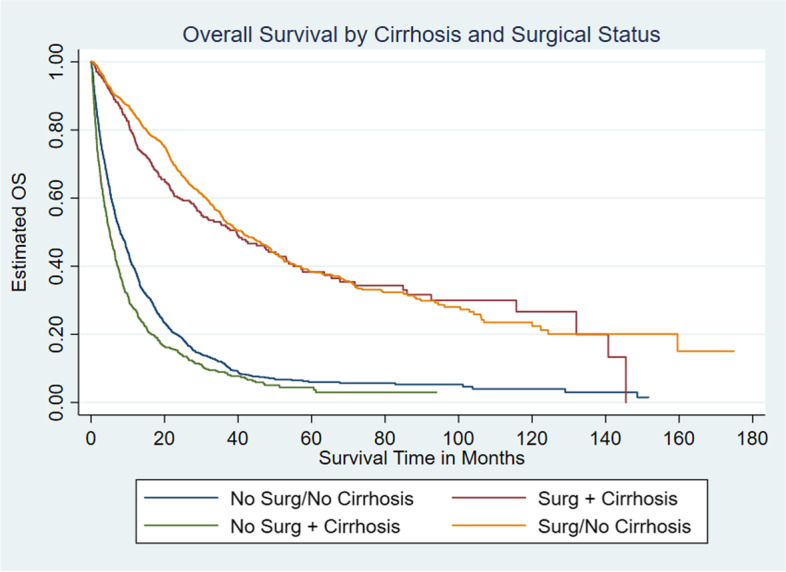
Kaplan-Meier Estimated Overall Survival (OS) among intrahepatic cholangiocarcinoma (CCA) by cirrhosis and surgical intervention status

**Fig. 4:**
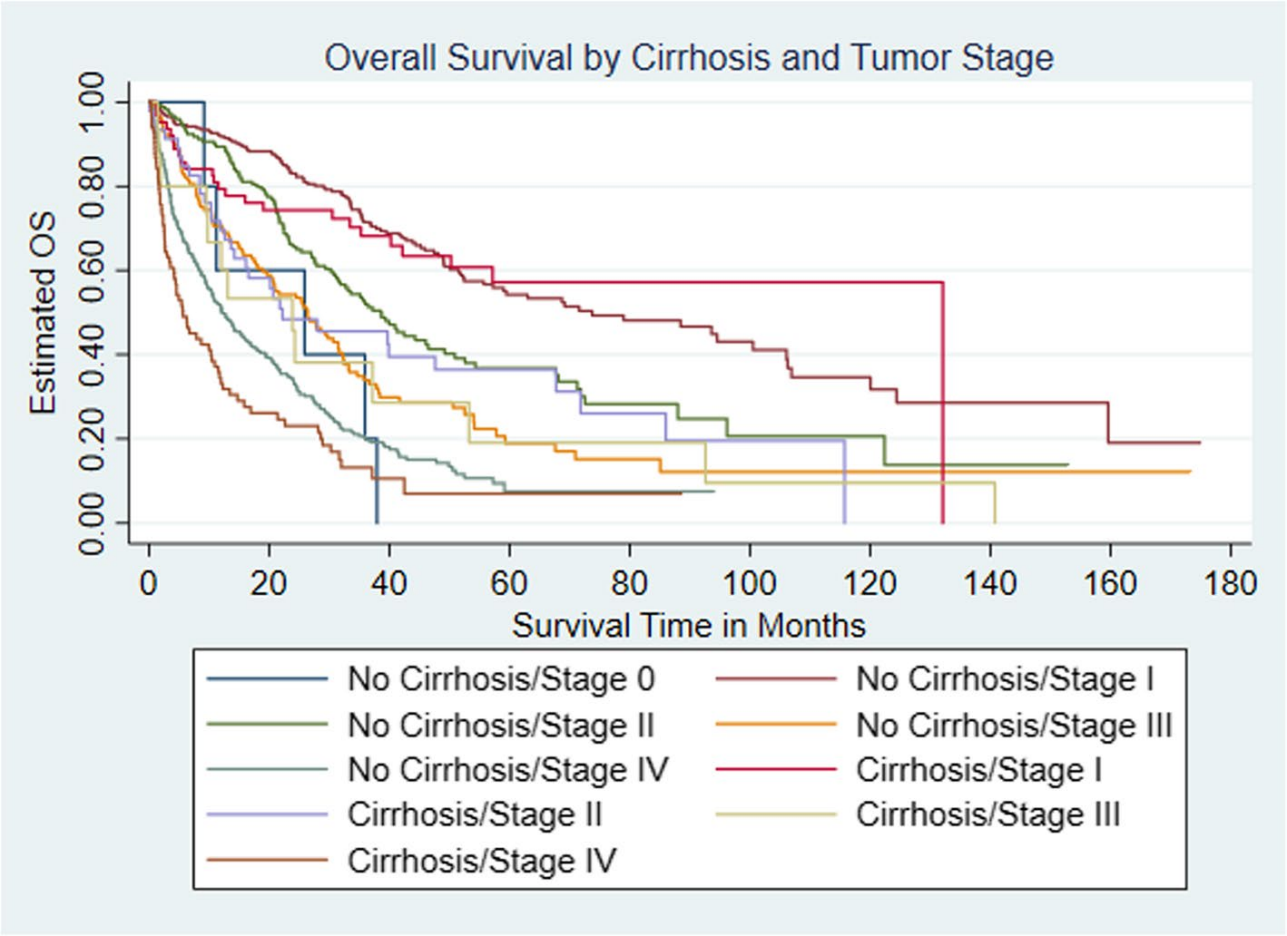
Kaplan-Meier estimated overall survival (OS) among intrahepatic cholangiocarcinoma (CCA) by cirrhosis and tumor stage

Among the entire population of CCA patients with known survival status, 84.4% (*n* = 39,406) died. Among iCCA patients with known survival status, 72.4% (*n* = 1564) of iCCA patients without cirrhosis patients died compared to 78.7% (*n* = 690) of iCCA patients with cirrhosis*. *iCCA patients with concomitant cirrhosis had a median OS half that of patients without cirrhosis (8.9 vs. 18.0 months, *P* < 0.001) (Fig. 2 and Table 4). We further examined median OS among iCCA patients with and without cirrhosis stratified by surgical intervention received and tumor stage. Among both the cirrhosis and non-cirrhosis cohorts, patients who underwent surgery had a significantly longer median overall survival (OS) time. However, results suggest a survival advantage for the non-cirrhosis cohort (41.2 months) compared to the cirrhosis cohort (39.7 months) (*p* < 0.001), with regards to surgical intervention (Fig. 3 and Table 5). When median OS stratified by tumor stage was assessed, results indicated that the cirrhosis cohort with Stage I tumor had the highest median OS time (132 months). However, the upper limit of the 95% confidence interval for the aforementioned cohort could not be estimated. Similarly, among the non-cirrhosis cohort, patients with Stage I tumor had the highest median OS time (73.7 months). Patients with the highest tumor stages among both the cirrhosis and non-cirrhosis cohorts had the lowest median OS (cirrhosis with stage IV: 5.6 months vs. non-cirrhosis with stage IV: 12.2 months) (*p* < 0.001). Patients with cirrhosis and stage IV disease had half the survival time of patients without cirrhosis and stage IV disease (Fig. 4 and Table 6).

**Table 4:** Median OS among patients with intrahepatic cholangiocarcinoma by presence of cirrhosis

Cirrhosis Status	Sample Size (n)	Median Overall Survival Time in Months (95% CI)
No Cirrhosis	2157	18.0 (16.5, 19.8)
Cirrhosis Present	873	8.9 (7.9, 10.3)
Total	3030	14.7 (13.4, 16.1)

**Table 5:** Median OS among patients with intrahepatic cholangiocarcinoma by presence of cirrhosis and surgical intervention status

Cirrhosis/Surgical Intervention Status	N	Median Overall Survival Time in Months (95% CI)
Cirrhosis Cohort		
Surgical Intervention	272	39.7 (28.9, 50.2)
No Surgical Intervention	594	5.1 (4.3, 5.8)
Non-Cirrhosis Cohort		
Surgical Intervention	982	41.2 (36.4, 46.4)
No Surgical Intervention	1163	8.0 (7.1, 9.3)
Total	3011	14.6 (13.4, 16.0)

**Table 6:** Median OS among patients with intrahepatic cholangiocarcinoma by presence of cirrhosis and tumor stage

Cirrhosis/Surgical Intervention Status	N	Median Overall Survival Time in Months (95% CI)
Cirrhosis Cohort		
Stage I	63	132.0 (42.1, NE)
Stage II	46	22.2 (13.6, 67.7)
Stage III	15	23.9 (1.9, 53.2)
Stage IV	83	5.6 (3.5, 10.4)
Non-Cirrhosis Cohort		
Stage 0^a^	–	25.9 (9.1, NE)
Stage I	260	73.7 (55.7, 106.1)
Stage II	173	38.6 (31.5, 46.4)
Stage III	129	26.4 (19.6, 31.5)
Stage IV	295	12.2 (9.7, 15.7)
Total^a^	–	30.2 (27.1, 32.7)

*NE* Not estimable

^a^Per NCDB user agreement pertaining to sample sizes less than 10, non-cirrhosis stage 0 sample size and total count are omitted

In univariate analyses using the KM method with log-rank tests, results consistently showed a survival advantage for non-cirrhotic patients. However, when a multivariate approach was used to assess survival outcomes and its association with the presence of cirrhosis among iCCA, there were no statistically significant associations found between cirrhosis and survival status (OR = 0.82, *p* = 0.405) (Table 7) or long-term survival (OR = 0.98, *p* = 0.933) (Table 7). In conclusion, while the initial results suggest that patients without cirrhosis had a survival advantage as measured by median overall survival in months, the presence of cirrhosis is not an independent prognostic factor of survival status or long-term survival.

**Table 7:** Multivariable logistic regression models assessing the presence of cirrhosis and its association with survival outcomes

	OR (95% CI)	*P*-Value	AOR (95% CI)	*P*-Value
Survival Status ^a^				
No Cirrhosis	Ref	Ref	Ref	Ref
Cirrhosis Present	0.71 (0.59, 0.86)	**< 0.001**	0.82 (0.52, 1.31)	0.405
Long-Term Survival ^b^				
No Cirrhosis	Ref	Ref	Ref	Ref
Cirrhosis Present	0.89 (0.75, 1.06)	0.182	0.98 (0.62, 1.55)	0.933

*OR* Odds Ratio, *AOR* Adjusted Odds Ratio, *95% CI* 95% Confidence Interval

Bold font denotes statistical significance at *P* < 0.05

^a^ Final multivariate model from stepwise backward selection adjusted for median income quartile, grade, and pathological stage. In the assessment of survival status, patients who died served as the reference group

^b^ Final multivariate model from stepwise backward selection adjusted for facility type, grade, and pathological stage. In the assessment of long-term survival, patients with survival less than 60 months after the date of diagnosis served as the reference group

## Discussion

Despite groundbreaking advances in the field of oncology over the last few decades, surgical resection remains the treatment of choice for all subtypes of CCA. However, early surgical treatment is often limited by the presence of concomitant cirrhosis. As known widely, surgical intervention in cirrhosis is associated with a risk of decompensation as defined by the development of ascites, variceal hemorrhage and hepatic encephalopathy and worsening jaundice. Cirrhosis not only complicates post-surgical recovery but also increases overall mortality for the patient, with 1 month and 3-month mortality rates being reported as high as 17 and 21% respectively [14].

The average life expectancy for a patient with decompensated cirrhosis is 2 years in comparison to 12 years for compensated cirrhosis [15]. Unfortunately, unlike in the case of hepatocellular carcinoma, liver transplantation as a treatment modality has been established for only a select group of patients with perihilar cholangiocarcinoma [16]. Patients with iCCA have however not shown to benefit from this intervention due to high rates of recurrence and poor long-term survival, with the 5-year patient survival varying from 0 to 42% [17]. As such, the need for a prognostic marker in the setting of iCCA is of prime importance to help guide treatment and goals of care.

The presence of cirrhosis in the setting of iCCA has been a contested topic in terms of its importance as a factor for prognostication with previous studies showing conflicting results. As early as 2011, a study of 132 patients with iCCA, of which 32% had concomitant cirrhosis, showed cirrhosis to be an independent factor for poor prognosis following surgical resection [18]. Another study done in 2017 with patient population of 106, of which 23.6% had concomitant cirrhosis, showed no difference in prognosis between the cirrhotic and non-cirrhotic arms [19]. A SEER (Surveillance, Epidemiology and End Results) database study done in 2020 with 512 patients showed that the presence of advanced fibrosis (defined by Ishak fibrosis score 5–6) was associated with worse cancer-specific survival across follow up periods (HR 1.49 (1.13–1.96, *p* = 0.005); HR 1.44 (1.14–1.83, *p* = 0.002) and HR 1.45 (1.15–1.83, *p* = 0.002) for 12, 36 and 60 months, respectively [20]. A recent multicenter retrospective study comparing outcomes of patients with liver cirrhosis undergoing liver transplant or surgical intervention in patients with iCCA or combined hepatocellular cholangiocarcinoma, found that survival improved after liver resection in patients with cirrhosis if tumor size was less than 5 cm [21]. Another retrospective analysis of 156 patients after surgical resection, the presence of cirrhosis did not have a significant impact on survival [22]. In another study of 184 patients published in 2020, cirrhosis did not have a significant difference in survival in iCCA patients (32 vs 33 months, *p*-value = 0.8) [23].

To our knowledge, this is one of the largest retrospective studies, examining a total of 3644 patients with iCCA using the NCDB data to analyze the impact of cirrhosis in patients with iCCA. After adjusting for socio-demographic and clinical characteristics, results showed that the presence of cirrhosis among iCCA patients was not associated with survival status or long-term survival, reflecting similar findings as some of the studies mentioned above. iCCA patients with cirrhosis had OR of 0.71, but when adjusted for median income quartile, grade and pathological stage, there was no statistical significance, with OR 0.82 (0.52–1.31, *p* = 0.405). Long term survival (survival more than 60 months after diagnosis) yielded similar results with no statistically significant differences.

Interestingly, after stratifying by surgical intervention, iCCA patients with cirrhosis benefitted the most from surgical intervention. When stratifying by stage, iCCA patients with cirrhosis exhibited lower median OS for stage IV disease compared to patients without cirrhosis. Among both the cirrhosis and non-cirrhotic cohorts, patients who underwent surgery had a significantly longer median overall survival (OS) time. In the cirrhosis cohort, median overall survival was 39.7 months for those who underwent surgical intervention, compared to 5.1 months for those who did not. Similarly, in the non-cirrhosis cohort, those who underwent surgical intervention had a median OS of 41.2 months compared to 8.0 months. Our results suggest a slightly higher survival advantage for the non-cirrhotic cohort (41.2 months) compared to the cirrhosis cohort (39.7 months) (*p* < 0.001) regarding surgical intervention. In terms of presence of cirrhosis by tumor stage, the cirrhosis cohort with stage IV had a median OS of 5.6 months, compared to non-cirrhosis cohort with stage IV disease, which had a median OS of 12.2 months, indicating that patients with cirrhosis and stage IV disease had half the survival of those with stage IV disease without cirrhosis.

Based on our data, cirrhosis status may not uniquely explain survival status or long-term survival, but rather other clinical characteristics/markers within the cirrhotic group may affect survival status. For example, as seen in Table 2, a lower proportion of iCCA patients with cirrhosis had tumor size greater than or equal to 5 cm (48.8% vs. 62.2%) – this is likely due to regular screening/imaging protocols in cirrhotics leading to detection of smaller tumors. Cirrhotics had a higher percentage of poorly differentiated or undifferentiated tumors and a higher percentage of stage 4 disease, lower percentage of surgical intervention and chemotherapy received (including multiagent chemotherapy) with higher contraindications to surgery due to risk factors, and lower percentage of lymphovascular invasion. Future research needs to be done to further disentangle possible associations (such as prospective cohort studies) especially considering the limitations of using a cancer registry.

Socio-demographic factors can contribute to cancer survival, as reported in recent studies. For example, studies have documented that race/ethnicity, income, type of insurance resulted in failure to administer recommended chemotherapy. As demonstrated by Barrera et al*,* studies using the NCDB for common malignancies such as breast, lung and colon cancer, found that the aforementioned characteristics could impact receipt of chemotherapy [24].

Hepatic surgical resection is recommended by the American association for the Study of Liver Diseases (ASSLD) and European Association for the Study of Liver (EASL) for early-stage hepatocellular carcinoma– Barcelona Clinic Liver Cancer staging system (BCLC) Stage 0/A, it is contraindicated if there is presence of clinically significant portal hypertension or decompensated cirrhosis. As such, in the absence of liver transplantation, the initiation of palliative care in such patients becomes paramount. However, previous data has shown palliative care services remain heavily underutilized in patients with advanced liver disease who are not candidates for liver transplant. A single center retrospective study of 102 patients showed that of all patients who had cirrhosis and were denied transplant candidacy, only 11% received a palliative care consultation. Our study corroborates this finding as well. Although there was no statistically significant difference in the utilization of palliative care between the two arms, the utilization of palliative care was low for both groups, 10.7% for the non-cirrhotic vs 9.9% for the cirrhotic group, indicating the need to address this gap in the future.

### Limitations

Misclassification of CCA subtypes based on ICD-O coding is a potential limitation in our study as previous studies have shown that perihilar CCA (pCCA) is frequently misclassified as iCCA instead of eCCA, which could lead to overestimation of iCCA incidence and misclassification of the data. For instance, in a study conducted by Welzel et al., 91% of pCCA were incorrectly coded as iCCA, resulting in overestimation of iCCA incidence by 13% and underestimation of eCCA by 15% [25]. Another potential limitation is the potential for selection bias as our study only included patients who underwent a liver biopsy to determine cirrhosis status. Only 3644 out of 33,160 iCCA patient underwent liver biopsy (10.9%). This population may exhibit systematic differences from those who did not have a liver biopsy. For example, those receiving a liver biopsy may have been more likely to be surgical candidates as liver biopsies are often performed intra-operatively at the time of resection. Moreover, with the use of a cancer registry, there is always potential for miscoding and potential for missing patient data.

## Conclusion

In our study of 3644 patients with iCCA who underwent liver biopsy during the time of staging, there was no statistically significant difference in survival status or long-term survival between iCCA patients with cirrhosis compared to those without cirrhosis. When stratifying for surgical intervention, both groups tended to have improved median OS when surgery was performed, with slight advantage of the non-cirrhotic arm. When stratifying for stage, stage IV cirrhotic iCCA patients tended to have worse median OS than non-cirrhotic iCCA patients.

## Data Availability

The authors confirm that the data supporting our findings are available within the article and within. For further details regarding the data, please contact the corresponding author.

## Abbreviations

CCA: Cholangiocarcinoma
HC-CCA: hepatocelluar-cholangiocellular carcinomas
CUP: Carcinoma of Unknown Primary
ECOG: Eastern Cooperative Oncology Group
ALP: Alkaline Phosphatase
PS: Performance Status
OS: Overall survival
